# Decoding Clinician Authorial Style: A Style-Informed Pipeline for Clinical Document Summary Generation with Large Language Models

**DOI:** 10.21203/rs.3.rs-9054955/v1

**Published:** 2026-03-26

**Authors:** Scott Zhao, Abbas Alili, Usman Afzaal, Muhammet F. Demir, Hao Lu, Padageshwar Sunkara, Metin N. Gurcan

**Affiliations:** Wake Forest University; Wake Forest University; The Ohio State University; Wake Forest University; Wake Forest University; Wake Forest University; Wake Forest University

## Abstract

Large language models (LLMs) can automate clinical document summary generation. However, even clinically accurate outputs often fail to reflect individual clinicians’ writing styles, leading to substantial post-editing. We examine this stylistic gap using a multi-author corpus of de-identified clinical summaries. We propose a style-informed generation framework that extracts clinician-specific stylistic features through LLM feedback and applies a Train→Generate paradigm to produce personalized clinical summaries. Conventional metrics (ROUGE, BERTScore, cosine similarity) largely failed to distinguish intra-author from inter-author writing patterns, while Jaro-Winkler and BLEU demonstrated limited sensitivity. Targeted LLM-guided feature extraction—emphasizing rhythm, narration, and sentence or list structure—improved authorship classification up to 73% of accuracy. In blinded clinician A/B testing, GPT-4-generated drafts were preferred less often than original notes, whereas the Gemini 2.5 Pro pipeline produced drafts preferred at rates comparable to, and in some cases exceeding, clinician-authored summaries. While inherent hallucination risks were noted, they were mitigated via high-fidelity prompt engineering and explicit adherence to source-only data constraints. These results suggest that style-informed generation can reduce the style gap and produce clinically acceptable clinical summaries that better align with the clinician’s voice.

## 1. Introduction

The hospital clinical summary serves as the primary clinical bridge for patient handoffs, acting as the definitive communication tool to ensure continuity of care between treatment teams and reduce the risk of avoidable readmissions^1,2^. During the precarious transition period covering the first 72 hours following a hospital stay, patients are uniquely vulnerable to adverse drug events and fragmented follow-up care due to a lack of or poor communication. This vulnerability comes due to the complexity of medical interventions and frequent transitions between care settings^2^. Ensuring the precision of this documentation is vital for patient safety. However, this necessity creates a significant administrative load on healthcare providers. In practice, generating a single clinical summary is a demanding process, requiring an average of 8.1 minutes for dictation and a median of 29.2 minutes for transcription and editing, often resulting in documents several pages in length^3^. This substantial time investment increases clinicians’ cognitive load, fueling documentation-related burnout and diverting essential time from direct patient care^4^.

Large language models (LLMs) offer a transformative solution to this administrative burden by automating the synthesis of fragmented patient data. Advanced architectures have demonstrated remarkable capabilities in semantic processing and clinical reasoning. Recent benchmarks even suggest that LLM-generated summaries can rival physician-authored texts in coherence and fluency^5^. Research by Ganzinger et al.^6^ and Oliveira et al.^7^ confirms that open-source models, when appropriately fine-tuned, can achieve high factual fidelity. However, the current body of literature predominantly operationalizes ‘quality’ through the lens of correctness. Studies focus primarily on mitigating hallucinations and ensuring data completeness^8^. While these results validate the feasibility of LLMs as drafting tools, they largely treat the clinical summary as a standardized informational output, emphasizing factual inclusion over clinical intent. This perspective overlooks the intrinsic variability in clinical documentation. Consequently, current models yield more generic narratives that, while factually accurate, fail to align with the attending physician’s distinct ‘voice’ and communicative preferences.

The inability of current LLMs to replicate individual writing styles represents a critical yet overlooked barrier to clinical adoption. This challenge shifts the research focus from simple data extraction to the more complex domain of provider-specific adaptation. While modern models are adept at synthesizing facts, they frequently suffer from a ‘homogenization effect’ which is hypothesized to be a byproduct of standard Reinforcement Learning from Human Feedback (RLHF) strategies that prioritize safety and uniformity over diversity^9,10^. This alignment process often homogenizes idiosyncratic vocabulary and professional shorthand, effectively ‘bleaching’ the nuanced clinical gestalt from the documentation^11,12^.

This lack of stylistic alignment imposes a secondary editing burden on clinicians^13^. Providers are forced not merely to verify facts, but to actively rewrite outputs to restore the subtle linguistic cues of diagnostic uncertainty and professional judgment—a process that can negate the time-saving benefits of automation^6^. These stylistic markers are not cosmetic; they are functional components of safe patient handovers, often signaling case complexity or urgency to receiving teams. Consequently, closing this gap requires moving beyond generic summarization prompts. It demands bespoke architectures (e.g., provider-specific in-context learning or few-shot style injection^13,14^) that enable the AI to align with the clinician’s established professional identity.

**Figure 1:**
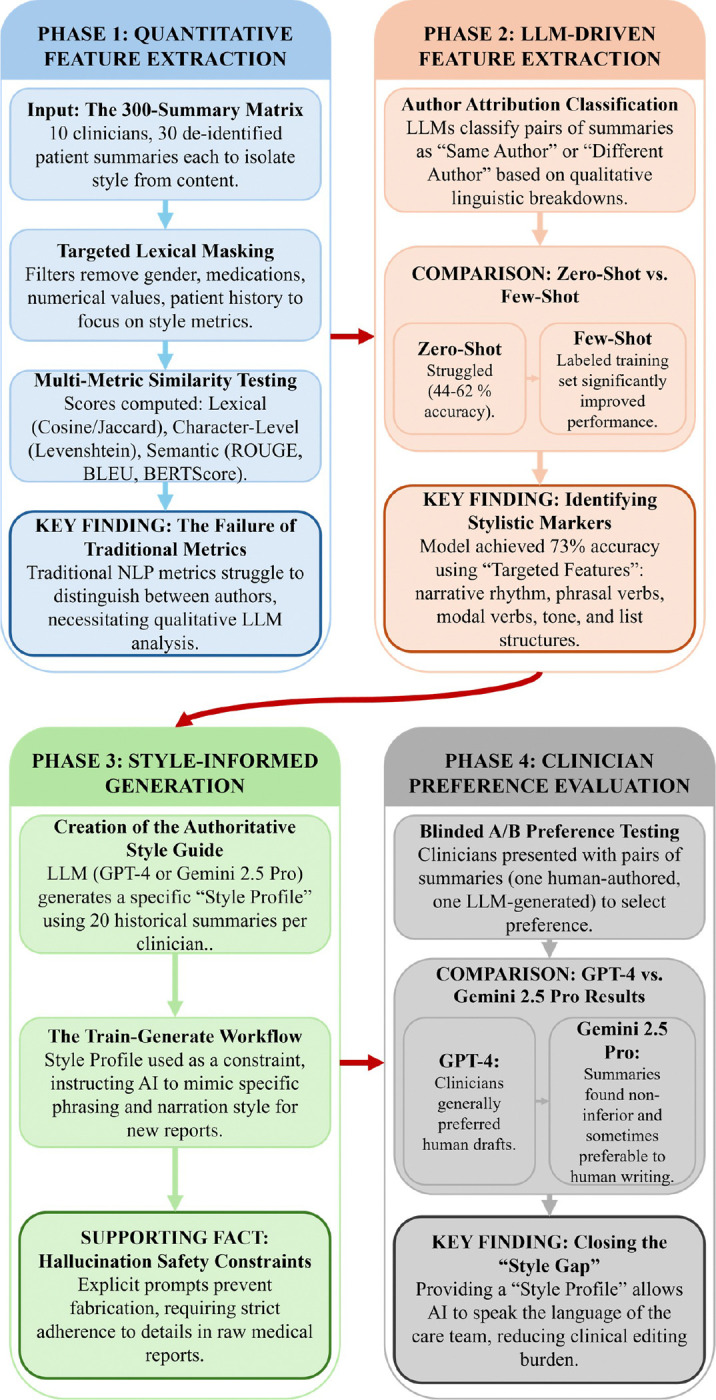
The Style-Informed Generation Framework This four-phase workflow illustrates the transition from traditional quantitative analysis to personalized clinical documentation. The process begins with Phase 1, evaluating the limitations of standard metrics through lexical masking and similarity testing. Phase 2 uses a feedback loop to identify high-dimensional linguistic markers that traditional metrics miss. Phase 3 implements these markers into a clinician-specific style guide for summary generation, while Phase 4 validates the output through blinded preference testing.

To address this gap, we propose a novel framework for **style-informed clinical summary generation** that goes beyond generic fidelity metrics. In this study, we first systematically evaluate the limitations of traditional quantitative similarity metrics (e.g., ROUGE, BLEU, BERTScore) in capturing authorial voice, demonstrating their insufficiency for differentiating between clinician-authored texts. Second, we introduce an **LLM-driven feedback loop** (Fig. 1) designed to extract high-dimensional qualitative features (e.g. narrative density, syntactic preferences, and hedging strategies) that correlate with authorship identity. Third, we utilize these identified stylistic markers to inform a custom generation pipeline using GPT-4 and Gemini 2.5 Pro, comparing zero-shot and few-shot approaches. Finally, we validate this framework through a blinded A/B preference study with attending clinicians, comparing their historical notes generated a year ago against our style-adapted generations. This work contributes to a foundational pathway for personalized clinical NLP, offering a reproducible methodology to reduce the editing efforts and enhance the clinical utility of automated documentation.

## 2. Methods

### 2.1. Study Design and Data Collection

We utilized a cross-sectional study design featuring a parallel corpus of clinical summaries to isolate authorial style from clinical content. Ten attending clinicians were recruited to review the charts of 30 unique, de-identified patients. Each clinician independently reviewed the same set of patient charts and authored a clinical summary. This design controlled clinical content while allowing stylistic variation across authors. This yielded a matrix of N = 300 ground-truth summaries, enabling direct “head-to-head” comparison of inter-annotator variability.

Our study design comprised four phases (see Fig. 1). The goal of *phase 1* of our methods was to extract quantitative features to identify similarities and differences in authorial style. This was done by evaluating a variety of computational similarity metrics on pairs of summaries written by the same or different clinicians. In *phase 2*, we used LLMs to explore more nuanced qualitative features, focusing specifically on the linguistic and stylistic elements of clinician writing. To assess Sthe usability of these features, we created an LLM classification task to determine whether pairs of written summaries could be successfully classified as written by the same author or different authors. This ideal set of features was then used in *phase 3* to drive stylistically informed LLM-generated hospital discharge summaries. Finally, in *phase 4*, we compared our LLM-generated summaries against clinician-written summaries. The available attending clinicians were each presented with 10 clinical reports and asked to indicate their preference for either their self-authored previously written summary or the stylistically informed LLM-generated summary, or both.

### 2.2. Phase 1: Quantitative Feature Extraction

To test the hypothesis that traditional NLP metrics are insufficient for capturing authorial style, we conducted a systematic similarity analysis. We defined two distinct similarity conditions:

1. **Intra-Author Similarity (**\varvec*S*_\varvec*i*\varvec*n*\varvec*t*\varvec*r*\varvec*a*_): Comparison of summaries written by the *same* clinician for *different* patients. High scores here indicate a consistent, template-driven style.
2. **Inter-Author Similarity (**\varvec*S*_\varvec*i*\varvec*n*\varvec*t*\varvec*e*\varvec*r*_): Comparison of summaries written by *different* clinicians for the *same* patient. High scores here indicate that the metric is capturing factual content rather than stylistic nuance.

We computed similarity scores, ranging from 0 for no similarity to 1 for complete similarity, using a suite of structural and semantic metrics:

- **Lexical Metrics**: Cosine Similarity^15^ and Jaccard Index^16^ to measure word-level overlap.
- **Character-Level Metrics**: Levenshtein Distance^17^, Jaro^18^, and Jaro-Winkler^19^ scores to capture formatting and abbreviation variances.
- **Semantic Metrics**: ROUGE (1, 2, L)^20^ and BLEU^21^ to assess n-gram recall/precision, and BERTScore to measure embedding-space semantic equivalence.

We refer to lexical and character-level metrics as **“simple similarity metrics,”** while embedding-based metrics are categorized as **“advanced semantic metrics.”** We hypothesized that an ideal style-sensitive metric would yield significant divergence between \varvec*S*_\varvec*i*\varvec*n*\varvec*t*\varvec*r*\varvec*a*_ and \varvec*S*_\varvec*i*\varvec*n*\varvec*t*\varvec*e*\varvec*r*_, where \varvec*S*_\varvec*i*\varvec*n*\varvec*t*\varvec*r*\varvec*a*_ would be high and \varvec*S*_\varvec*i*\varvec*n*\varvec*t*\varvec*e*\varvec*r*_ would be low.

However, in practical clinical settings, patient conditions and care trajectories differ substantially. As a result, summaries written by the same clinician for different patients are expected to share limited surface similarity, yielding lower \varvec*S*_intra_. Conversely, summaries written by different clinicians for the same patient necessarily describe the same diagnoses, procedures, and hospital course, leading to higher \varvec*S*_inter_. Therefore, we tested the effect of applying a targeted lexical masking to the written summaries on the computation of similarity scores. To do this, we tokenized the summaries into individual words and removed predefined sets of words and phrases to reduce the influence of patient-specific context on similarity comparisons. For inter-author similarity, \varvec*S*_\varvec*i*\varvec*n*\varvec*t*\varvec*e*\varvec*r*_, we removed gendered terms (e.g., “male”, “female”), medications, numerical values (e.g., ages, dates), and common basic phrases (e.g., “year old”, “hospitalist”). For intra-author similarity, \varvec*S*_\varvec*i*\varvec*n*\varvec*t*\varvec*r*\varvec*a*_, we removed gendered terms, medications, numerical values, and additionally, patient medical history and symptoms. Because when evaluating \varvec*S*_\varvec*i*\varvec*n*\varvec*t*\varvec*e*\varvec*r*_, doctors’ descriptions of medical history and symptoms may reflect stylistic preference. In contrast, when evaluating \varvec*S*_\varvec*i*\varvec*n*\varvec*t*\varvec*r*\varvec*a*_, differences in medical history and symptoms primarily reflects differences in patient contexts rather than contextual authorial style.

To evaluate the effectiveness of each similarity metric and the masking, two hypothesis tests were performed on each metric:

1. Comparing \varvec*S*_\varvec*i*\varvec*n*\varvec*t*\varvec*e*\varvec*r*_ and \varvec*S*_\varvec*i*\varvec*n*\varvec*t*\varvec*r*\varvec*a*_ *before* applying any masking, and
2. Comparing \varvec*S*_\varvec*i*\varvec*n*\varvec*t*\varvec*e*\varvec*r*_ and \varvec*S*_\varvec*i*\varvec*n*\varvec*t*\varvec*r*\varvec*a*_ *after* applying appropriate masking.

Each hypothesis test was performed using a two-sample t-test, and significance was determined if the associated p-value was less than 0.05. Because we aimed to find similarity metrics where \varvec*S*_\varvec*i*\varvec*n*\varvec*t*\varvec*r*\varvec*a*_ was greater than \varvec*S*_\varvec*i*\varvec*n*\varvec*t*\varvec*e*\varvec*r*_, we used a one-sided alternative hypothesis (H_A_ : \varvec*S*_\varvec*i*\varvec*n*\varvec*t*\varvec*e*\varvec*r* <_ \varvec*S*_\varvec*i*\varvec*n*\varvec*t*\varvec*r*\varvec*a*_) for each hypothesis test.

### 2.3. Phase 2: LLM-Driven Feature Extraction

Given the hypothesized limitations of quantitative metrics, we developed a qualitative feedback loop using Large Language Models (LLMs) to identify high-dimensional stylistic features (Fig. 1).

To isolate the specific linguistic features that define a clinician’s style, we employed a two-stage classification strategy using Large Language Models (LLMs). We considered three possible labels for pairs of summary data:

1. **SD**: two summaries written by the same author about different patients,
2. **DS**: two summaries written by different authors about the same patient, and
3. **DD**: two summaries written by different authors about different patients.

Before generating new content, the model was evaluated on its ability to distinguish summary pairs, where “SD” is classified as *“same author”* and “DS” and “DD” are classified as *“different authors”*. This was done by computing the overall accuracy and accuracy within each of the three possible summary pairs.

#### Zero-Shot Classification

We established a baseline using a Zero-Shot classification approach^22, 23^, utilizing the entire dataset without prior training examples or task-specific training. The model was presented with paired clinical summaries and given the following instructions

“For each of the entry pairs, verify if the two input texts were written by the same author using qualitative, detailed linguistic breakdown. Analyze the writing styles of the input texts, disregarding differences in topic and content. Reasoning based on linguistic features including but not limited to phrasal verbs, modal verbs, rare words, affixes, quantities, tone, and abbreviation usage. Note that some pairs are written by the same author, and some pairs are written by different authors.”

#### Few-Shot Classification (Train-Test Split):

To improve classification accuracy, we implemented a few-shot classification approach^24, 25^ using a train-test split. In this setting, the model was first provided with a labelled training set consisting of summary pairs annotated as either authored by the *same* clinician or by *different* clinicians. This in-context learning allowed the model to learn corpus-specific stylistic cues before generating predictions on a held-out test set. We evaluated multiple train-test split ratios (100:100, 120:80, 140:60, 160:40). ). The model was given the following instructions:

“Take the attached data as the training data set and develop a predictor using qualitative detailed linguistic features to predict whether two input texts are written by the same author. Analyze the writing styles of the input texts, disregarding differences in topic, content, and grammatical errors.”

For the test set, we evaluated three distinct **prompt**^26, 27^ **endings** to determine the optimal level of instruction specificity:

- **Prompt Ending A (Baseline)**: An uninformative instruction.

“For each input text pair, verify if they were written by the same or different author using the previously developed predictor.”

- **Prompt Ending B (Broad Qualitative)**: Focused on general linguistic features.

“Using the previously developed predictor, verify if the two input texts were written by the same author using qualitative detailed linguistic authorship breakdown. Analyze the writing styles of the input texts, disregarding differences in topic and content and grammatical errors. Reasoning based on linguistic features including sentence and list structure, phrasal verbs, modal verbs, affixes, tone, and abbreviation usage.”

- **Prompt Ending C (Targeted Feature)**: Narrowed focus based on features identified during preliminary qualitative extraction (e.g., rhythm, narration style).

“Using the previously developed predictor, verify if the two input texts were written by the same author using qualitative detailed linguistic authorship breakdown. Analyze the writing styles of the input texts, disregarding differences in topic and content and grammatical errors. Reasoning focused on writing style elements of sentence and list structure, phrasing, tone, rhythm, and narration style.”

### 2.4. Phase 3: Style-Informed Generation

Based on the superior performance (see Results 3.2) of the targeted feature extraction (Prompt Ending C) in our testing classification accuracy, we advanced to the generation phase. This workflow utilized a “Train-Generate” paradigm to create new summaries that mimicked a specific provider’s style. Early attempts at summary generation led to hallucinations, as clinicians identified fabricated and false clinical information in LLM summaries that contradicted the original text of the clinical summary. To address this issue, we employed a strong and explicit prompting strategy to create an authoritative style guide. This style guide outlined how to capture provider-specific stylistic features and what information the LLM could and couldn’t use in its summary generation.

#### Training Phase (Style Profiling)

For each target clinician, we allocated 20 previously authored summaries to a training set for in-context learning. In this phase, GPT-4 and Gemini 2.5 Pro were instructed to analyze these examples to create an authoritative style guide for that specific user.

“Use the attached data as a training data set to analyze the writing style of the author writing the summaries. Focus on writing style elements of sentence and list structure, phrasing, tone, rhythm, and narration style. Use this analyzed writing style as the authoritative style guide for generating all future summaries.”

#### Generation Phase

In the final stage, the model generated new summaries for held-out reports (N = 10 per provider). The prompt included strict safety constraints to prevent hallucinations, explicitly requiring the model to adhere to the previously created authoritative style guide while using only the facts presented in the new report.

“Use the identified writing style of this author developed from the training data set to generate a one paragraph stylistically consistent summary for the report below labeled ‘Report:’. When generating the summary for the new report, your output must strictly follow the stylistic conventions of the training summaries, including sentence and list structure, phrasing, tone, rhythm, and narration style. Be sure that generated summaries are concise like the original summaries in the training data set and concentrate on the specific content mentioned in the report to make them informative and clinically focused. Do not fabricate or infer any information. Only summarize details explicitly stated in the report. If information is missing, leave it out — do not guess or create new clinical facts.”

### 2.5. Phase 4: Clinician Preference Evaluation (A/B Testing)

To validate clinical utility, we conducted a blinded A/B preference study^28^ with practicing clinicians. Clinicians were presented with pairs of summaries for cases they had previously documented (one Human-authored, which they had previously written, one GPT-4 or Gemini 2.5 Pro generated). For each pair, clinicians were asked to select a preference: “Prefer Human,” “Prefer LLM,” or “Looks the Same”. Statistical significance was assessed using a one-sample proportion hypothesis test ($H_{0}:p=0.5$), treating “Looks the Same” as a neutral outcome weighted equally between the two categories.


$$\overset{ˆ}{p}=\frac{PreferHuman\;+\;0.5*LookstheSame}{TotalResponses}$$

This four-phase workflow illustrates the transition from traditional quantitative analysis to personalized clinical documentation. The process begins with Phase 1, evaluating the limitations of standard metrics through lexical masking and similarity testing. Phase 2 uses a feedback loop to identify high-dimensional linguistic markers that traditional metrics miss. Phase 3 implements these markers into a clinician-specific style guide for summary generation, while Phase 4 validates the output through blinded preference testing.

## 3. Results

### 3.1. Limitations of Traditional Quantitative Metrics

**Figure 2:**
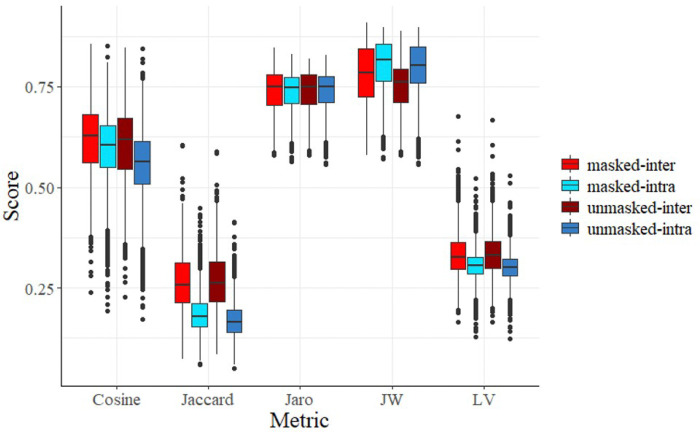
Effectiveness of Simple Similarity Metrics on Masked and Unmasked Data For each metric, we computed similarity scores between all possible pairs of written summaries in the masked and unmasked case. We used this data to perform hypotheses tests on the difference between S_inter_ and S_intra_. Jaro-Winkler abbreviated to JW, Levenshtein abbreviated to LV.

An ideal similarity metric should have high intra-author scores ($S_{intra}$) and low inter-author scores $\left(S_{inter}\right)$. The simple similarity metrics (lexical and character-level) demonstrated a limited ability to isolate authorial style from patient-specific content. As shown in Fig. 2, for the **Cosine, Jaccard, and Levenshtein** metrics, the inter-author similarity was consistently higher than the intra-author similarity. The high $\left(S_{inter}\right)$ scores indicate that these metrics primarily track shared medical terminology and factual overlap, rather than unique phrasing. Table 1 highlights that **Jaro-Winkler (JW)** was the only simple metric to achieve statistical significance in both masked (p=4.43e-28) and unmasked (p=5.76e-122) conditions. The relative success of Jaro-Winkler suggests it is more sensitive to “character-level” stylistic habits, such as specific ways of abbreviating or consistent sentence patterns, which remain consistent for an author even when the medical content changes.

**Figure 3:**
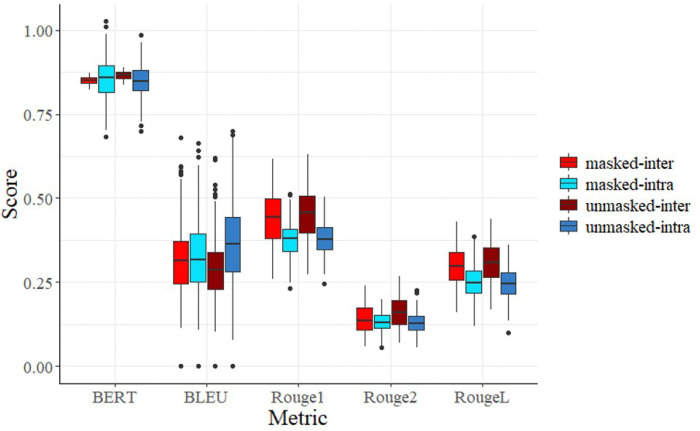
Effect of Masking on Advanced Similarity Metrics For each metric, we computed similarity scores between all possible pairs of written summaries in the masked and unmasked cases. We used this data to perform hypothesis tests on the difference between S_inter_ and S_intra_. BERTScore is abbreviated to BERT.

The advanced semantic metrics (ROUGE, BLEU, BERTScore) were expected to better capture “clinical intent,” yet they largely mirrored the failures of lexical metrics. As illustrated in Fig. 3, **ROUGE-1, ROUGE-L, and BERTScore** failed to produce a significant difference between $S_{intra}$ and $S_{inter}$. In fact, the *pvalues* for ROUGE-1 and ROUGE-L were 1.0, indicating that these metrics computed higher scores for $S_{inter}$ than $S_{intra}$, the exact opposite of what we wanted to capture in our similarity metrics. Interestingly, applying targeted lexical masking (removing medications, dates, and gendered terms) did not made substantial change in these metrics. Even after removing patient-specific facts, the BERTScore embeddings still gravitated toward the shared medical narrative rather than the authorial voice.

Table 2 shows that BLEU achieved statistical significance (p=2.55e-16) only in the unmasked state. This suggests that BLEU’s sensitivity to specific n-gram sequences is heavily tied to the raw clinical data, and once that data is masked, its ability to detect authorial voice vanishes.

### 3.2. Efficacy of LLM-Driven Feature Extraction

Transitioning to qualitative feature extraction using LLMs yielded a marked improvement in authorship attribution, validating the hypothesis that style is better defined by high-dimensional linguistic features than by n-gram overlap.

- **Zero-Shot Baseline**: As shown in Table 3, the Zero-Shot baseline struggled significantly, with overall accuracy fluctuating between 44.67% and 62%. Without specific stylistic training, the model often performed only slightly better than a random guess when trying to identify if two summaries were written by the same author. The model showed extreme variance in identifying “Same Author” (SD) versus “Different Author” (DS/DD) pairs across different trials. In some trials, it over-identified same-author pairs, while in others, it did the opposite, indicating a lack of a stable internal stylistic capture.
- **Few-Shot & Prompt Engineering**: Results changed dramatically with the introduction of these methods. Specifically, Prompt Ending C which focused on rhythm, narration, and sentence/list structure, achieved the highest performance. Utilizing a 100:100 train-test split, the model reached an overall accuracy of 73%. Most notably, this approach achieved 80% accuracy in correctly identifying “Same Author” (SD) pairs. This suggests that once the LLM was instructed to look for specific high-dimensional markers like narrative rhythm and hedging strategies, it could successfully recognize the professional identity of the clinician.

### 3.3. Clinician Preference and Style Replication (A/B Testing)

The ultimate validation of the framework was the blinded A/B preference study. Results indicated a performance divergence between model architectures (GPT-4 vs. Gemini 2.5 Pro), with Gemini demonstrating stronger style replication capabilities.

- **GPT-4 Performance**: In the GPT-4 arm, clinicians retained a preference for human-authored summaries. Clinicians preferred the Human draft in 57 responses compared to 24 responses for the LLM. With a calculated p-value of 1.0 (Table 5) and a preference proportion (p) of 0.6833, the results failed to achieve statistical significance, indicating that GPT-4 did not sufficiently capture the unique nuances of the provider’s style.
- **Gemini 2.5 Pro Performance**: The Gemini model demonstrated superior style alignment. Clinicians showed a slight preference for the LLM-generated draft (43 responses) over their own historical notes (35 responses), with 12 responses rated as indistinguishable. The hypothesis test yielded a p-value of 0.4606 for Gemini. This suggests that the Gemini-based pipeline, informed by the authorial voice, generated summaries that were noninferior to the clinician’s original documentation. In some cases, the LLM generated clinical summaries were preferred over the clinician-authored notes. However, these differences did not reach statistical significance.

This comparative visualization displays the results of the blinded preference study for both model architectures. The data indicate the number of responses in which clinicians preferred their original human-authored notes, the LLM-generated drafts, or found the two to be indistinguishable. The results show a notable performance divergence, with Gemini 2.5 Pro achieving a higher rate of style alignment and clinician preference than GPT-4.

## 4. Discussion

Our findings challenge the prevailing assumption in medical NLP that factual correctness is the sole proxy for clinical utility. While prior studies demonstrate that LLMs achieve high semantic fidelity, our quantitative analysis reveals that traditional metrics—such as ROUGE, BLEU, and BERTScore—are fundamentally ill-suited for measuring stylistic concordance. As shown in our “Masking Effect” analysis, these metrics failed to reliably distinguish between clinical summaries written by different clinicians for the same patient. This confirms that high n-gram overlap captures the **content** of the medical case but fundamentally misses the **style** of the provider. For the clinical community, this implies that a high-scoring AI summary may still feel alien to the signing clinician, effectively “bleaching” the nuanced clinical gestalt and professional identity from the document. This perpetuates the editing load as providers are forced to rewrite accurate but tonally mismatched drafts. These findings suggest that stylistic personalization may represent a critical next step in clinical documentation automation.

To overcome the “homogenization effect” typical of RLHF-aligned models—which prioritize uniform, safe outputs over diverse authorial voices—this study introduces a **Style-Informed Generation Framework**. By shifting from zero-shot prompting to a “Train-Generate” pipeline, we successfully extracted high-dimensional features (such as narrative rhythm and hedging strategies) that elude traditional statistical detection methods. The efficacy of this approach was evidenced by a jump in authorship attribution accuracy to **73%** with targeted feature extraction, compared to the near-random performance of zero-shot baselines. When the LLM is explicitly constrained to a stylistic profile derived from historical data, it begins to convey the authorial voice of the care team, producing drafts that are often indistinguishable from human writing.

A critical finding of this study is the divergence in performance between model architectures. While GPT-4 produced fluent text, clinicians largely preferred their own human-written notes. In contrast, Gemini 2.5 Pro appeared more responsive to style-profile constraints in this experimental setting, with 43 clinician preferences compared to 35 for human drafts. This variance likely stems from how each model weights long-context instructions versus its underlying safety alignment. Gemini’s ability to generate “non-inferior” summaries suggests it may be better suited for applications requiring high-fidelity style transfer in-context learning.

### Limitations

While our framework mitigated hallucinations through strict prompt constraints, the safety-critical nature of clinical documentation requires ongoing verification. The small sample size (N = 10) and the use of static (“frozen”) Style Profiles limited an immediate generalizability. Future research should expand the cohort size beyond ten clinicians to improve the generalizability of these findings. The current approach should transition to dynamic models that evolve in real-time as a clinician’s documentation style changes over their career. Additionally, investigations into architectural differences are needed to determine why specific models, such as Gemini 2.5 Pro, demonstrate superior adaptability to in-context style constraints. Finally, future studies should employ downstream impact metrics such as clinician vs LLM time difference to provide a direct quantitative measure of how much the editing load is reduced in active clinical workflows.

### Conclusion

The integration of Large Language Models into electronic health records offers a path to mitigate clinician burnout, but only if the technology aligns with users’ professional identity. We demonstrate that while current models are factually capable, they face a “Style Gap” that traditional metrics fail to detect. By implementing a Style-Informed Feedback Loop, we provide a reproducible proof-of-concept for personalized clinical documentation. This approach moves the field closer to an AI assistant that not only “summarize,” but also aligns with the stylistic conventions of the clinical author, ultimately reducing administrative burden and allowing clinicians to return to patient care.

## Figures and Tables

**Figure 4:**
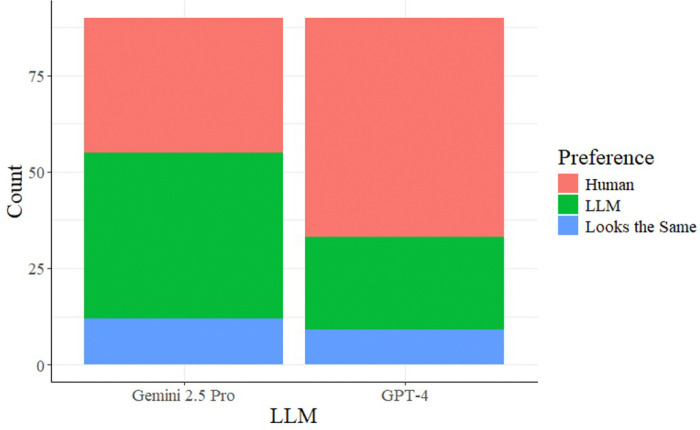
Distribution of Clinician Preferences in A/B Testing This comparative visualization displays the results of the blinded preference study for both model architectures. The data indicate the number of responses in which clinicians preferred their original human-authored notes, the LLM-generated drafts, or found the two to be indistinguishable. The results show a notable performance divergence, with Gemini 2.5 Pro achieving a higher rate of style alignment and clinician preference than GPT-4.

**Table 1:** **P-values of Hypothesis Tests on difference of** \varvec*S*_\varvec*i*\varvec*n*\varvec*t*\varvec*e*\varvec*r*_ **and** \varvec*S*_\varvec*i*\varvec*n*\varvec*t*\varvec*r*\varvec*a*_ **for Simple Metrics** For each metric, we performed two one-sided t-tests: 1) masked vs. masked and 2) unmasked vs. unmasked to evaluate if there is a significant difference between and. The p-values for these hypothesis tests are reported in the table.

Cosine	Masked	Unmasked
	1	1
Jaccard	1	1
Levenshtein	1	1
Jaro	0.846	0.318
Jaro-Winkler	4.43e-28	5.76e-122

**Table 2:** **P-values of Hypothesis Tests on the difference of** \varvec*S*_\varvec*i*\varvec*n*\varvec*t*\varvec*e*\varvec*r*_ **and** \varvec*S*_\varvec*i*\varvec*n*\varvec*t*\varvec*r*\varvec*a*_ **for Advanced Metrics** For each metric, we performed two one-sided t-tests: 1) masked vs. masked and 2) unmasked vs. unmasked to evaluate if there is a significant difference between and. The p-values for these hypotheses tests are reported in the table.

BERTScore	Masked	Unmasked
	0.177	1
BLEU	0.304	2.55e-16
Rouge1	1	1
Rouge2	0.980	1
RougeL	1	1

**Table 3:** Performance of Zero-Shot LLM Author Attribution This table displays the baseline classification results across six independent trials using a zero-shot approach without prior stylistic training. Accuracy scores represent the model's ability to distinguish between same-author pairs (SD) and different-author pairs (DS and DD). The results highlight the high variance and limited reliability of zero-shot models in identifying authorial signatures without specific training examples. Each row represents a trial with 150 total summary pairs, with 50 from each label.

Overall (%)	SD Label (%)	DS Label (%)	DD Label (%)
44.67	78	30	26
44.67	62	32	40
55.33	60	38	68
58	48	54	72
61.33	28	78	78
62	18	82	86

**Table 4:** Efficacy of Few-Shot Learning and Targeted Prompting This table compares the classification accuracy of three distinct prompt specificity levels using a 100:100 train-test split. The data demonstrates the significant performance improvement achieved through few-shot learning, particularly with Prompt 3 (Targeted Feature Extraction). This specific prompt reached a peak overall accuracy of 73% and an 80% success rate in identifying clinician-specific signatures, validating the use of high-dimensional linguistic markers for style replication. Each row represents a trial with 200 total summary pairs with 100 SD, 50 DS, and 50 DD.

Prompt	Overall (%)	SD Label (%)	DS Label (%)	DD Label (%)
1	46	46	48	35
2	47	44	52	50
3	73	80	60	65

**Table 5:** Statistical Significance of Clinician Preferences This table reports the outcomes of the one-sample proportion hypothesis test used to evaluate model performance. The p-values indicate whether the observed preference for a draft significantly deviated from the neutral baseline of 0.5. Results show that while GPT-4 results favored human drafts, the Gemini 2.5 Pro results indicated non-inferiority, as the preference difference was not statistically significant.

LLM	$\backslash \hat{\text{varvec}p}$	p-value
GPT-4	0.6833	1
Gemini 2.5 Pro	0.4556	0.4606

## Data Availability

The datasets generated and analyzed during the current study are not publicly available due to IRB restrictions at Wake Forest University School of Medicine (IRB00127840) regarding the protection of sensitive clinical data. However, de-identified data can be made available from the corresponding author upon reasonable request and following the execution of a formal Data Use Agreement (DUA) to ensure ethical and legal compliance.
